# Supplementary material for: Chromothripsis during telomere crisis is independent of NHEJ, and consistent with a replicative origin
Source: Genome Res. 2019 May;29(5):737–49. doi: 10.1101/gr.240705.118 (PMC6499312; doi:10.1101/gr.240705.118)
Supplement: Supplemental Material [file supp_gr.240705.118_Supplemental_file_1.zip › contigs/annotated_contigs/DB51/contig.3.DB51_length_714_mean_cov_7.60924369748.docx]

**DB51_length_714_mean_cov_7.60924369748**

```
CG|CCTGCGTTGGCCTCGAGCCCCCCCTCCCAGCCTTGTTCTATCTCTTTTTTCGTTAATGTGTCCTTCTTCACAGCCTGTTTAGTGCC
   >chr20:23414292-23414561 + E=6e-126
ACGTTTCACAATTTTGTATTGGTTTTTTTTTTAGAGGTGGGGTCTTGCTGTGTTGCCCAGGCTGATCTCGAACTCCTGAGCTCAAGTGT

CTTCAGCCTTGGCCTCCCAAAGTGTTGGCATTACAGGTATGAGCTATCACATGTGGTTTGAGTTTTGTGCTTTTTTTGGTTGGTGATTT

TGCTG|TTATTATGACT|ACTTATAAGTGAAAACATGCGGTATTTGGTTTTCTGTTCCTGCATTAATTCACTTAGG|ATC|ATCATTCT
                 >chr20:23391053-23391114 - E=6e-25 p=2e-03                >chr20:234
CAATAAACTATCGCAAAAACAAAAAACCAAACACCGCATATTCTCACTCACAGGTGGGAATTGAACAATGAGAACACATGGACACAGGA
58313-23458687 - E=3e-207
AGGGGAACATCACACTCTGGGGCCTGTTGTGGGGTGGGGGGAGAGGGGAGGGATAGCATTGGGAGATATACCTAATGCTAGATGACGAG

TTAGTGGGTGCAGCGCACCAGCATGGCACATGTATACATATGTAACTAACCTGCACAATGTGCACATGTACCCTAAAACTTAAAGTATA

ATAATAATAATAATAAATCATTGTCCACGTCTGTCAAGAAGAATTATACAAACAGTTGCTAAGTGTTTTCTTTTCATGAATTTTTTTAA

AAGGCAG
```
